# Exaggerated trans-membrane charge of ammonium transporters in nutrient-poor marine environments

**DOI:** 10.1098/rsob.220041

**Published:** 2022-07-13

**Authors:** Matthew Kellom, Stefano Pagliara, Thomas A. Richards, Alyson E. Santoro

**Affiliations:** ^1^ Department of Ecology, Evolution, and Marine Biology, University of California, Santa Barbara, CA, USA; ^2^ Living Systems Institute and Biosciences, University of Exeter, Exeter, Devon EX4 4QD, UK; ^3^ Department of Zoology, University of Oxford, 11a Mansfield Road, Oxford OX1 3SZ, UK

**Keywords:** competition, SAR11, SAR86, Thermoproteota, Thaumarchaea, membrane transport

## Abstract

Transporter proteins are a vital interface between cells and their environment. In nutrient-limited environments, microbes with transporters that are effective at bringing substrates into their cells will gain a competitive advantage over variants with reduced transport function. Microbial ammonium transporters (Amt) bring ammonium into the cytoplasm from the surrounding periplasm space, but diagnosing Amt adaptations to low nutrient environments solely from sequence data has been elusive. Here, we report altered Amt sequence amino acid distribution from deep marine samples compared to variants sampled from shallow water in two important microbial lineages of the marine water column community—Marine Group I Archaea (Thermoproteota) and the uncultivated gammaproteobacterial lineage SAR86. This pattern indicates an evolutionary pressure towards an increasing dipole in Amt for these clades in deep ocean environments and is predicted to generate stronger electric fields facilitating ammonium acquisition. This pattern of increasing dipole charge with depth was not observed in lineages capable of accessing alternative nitrogen sources, including the abundant alphaproteobacterial clade SAR11. We speculate that competition for ammonium in the deep ocean drives transporter sequence evolution. The low concentration of ammonium in the deep ocean is therefore likely due to rapid uptake by Amts concurrent with decreasing nutrient flux.

## Graphical abstract

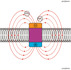

## 1. Introduction

Competition for resources is a fundamental driver of evolution [1–3]. Microbes living in low-nutrient environments, such as the open ocean, are under pressure to outcompete their neighbours, vying for limited resources [1,2,4]. Proteins that allow the acquisition and utilization of substrates at low concentrations may thus be under strong selection pressure. However, diagnosing quantitative variation in substrate affinity from sequence data alone can be challenging [5], inhibiting our ability to interpret competition between uncultivated microbes from metagenomic data.

Membrane-bound transporters are protein complexes that facilitate the flux of nutrients into the cytoplasm. Transporter proteins that create a channel for substrate transit through the membrane will often have protein regions that extend towards the interior or the exterior of the cell membrane, facilitating nutrient selection and uptake [6–8]. Such protein regions are subject to distinct selective pressures to maximize function given environmental, ecological and physiological constraints. This balance between function, physiology and environment determines marine microbial distribution by restricting growth of populations that cannot compete at low substrate concentrations [4] and results in genome-wide adaptations to elemental scarcity, particularly nitrogen [9–11]. To meet metabolic demands and remain competitive, microbes that live in low nutrient conditions are predicted to have transporter proteins with optimal affinity for their substrate [12,13], yet there is no clear demonstration of how such ecological circumstances have driven transporter sequence evolution.

At extracellular concentrations of ammonium less than 1 mM, microbes require transport systems such as ammonium transporters (Amts) [14]. Microbial Amts belong to the methyl-ammonia permease (MEP) family of transporter proteins with homologues found in all three domains of life [15]. Crystal structures were first reported for the AmtB protein of *Escherichia coli* [16,17], and the Amt-1 protein of *Archaeoglobus fulgidus* [18]. These proteins consist of 11 membrane-bound ɑ-helices with cytoplasm and periplasm extensions, forming individual channels in a trimer quaternary structure [18–20]. Amts have a history of uncertainty surrounding the mechanism of passive or active transport, as well as the specificity for ammonia (NH_3_) and/or ammonium (NH_4_^+^) [17,21–25]. Recent experiments describe Amt as a NH_4_^+^/H^+^ symporter, actively transporting primarily NH_4_^+^ with some transport of methylammonium and limited NH_3_ passage [26–28]. The preference of NH_4_^+^ is predicted from models due to the net negative charge of the periplasm extensions of the transporter protein, with NH_3_ passing through the transporter channel via deprotonation at a periplasm-facing active site and reprotonation/release at a cytoplasm-facing site [16,26,29,30]. The Amt active site at which NH_4_^+^ binds in the periplasm has been defined by a highly conserved ‘phenylalanine-gate’ motif, along with tryptophan and serine residues in AmtB [16,17,24]. During the transport of NH_4_^+^, the deprotonation-mediated proton concentration increase (and pH decrease) in the periplasm may further an acid-trap mechanism that accumulates NH_4_^+^ in the periplasm [31], demonstrating the importance of electrochemical properties to the function of these proteins.

**Figure 1.**
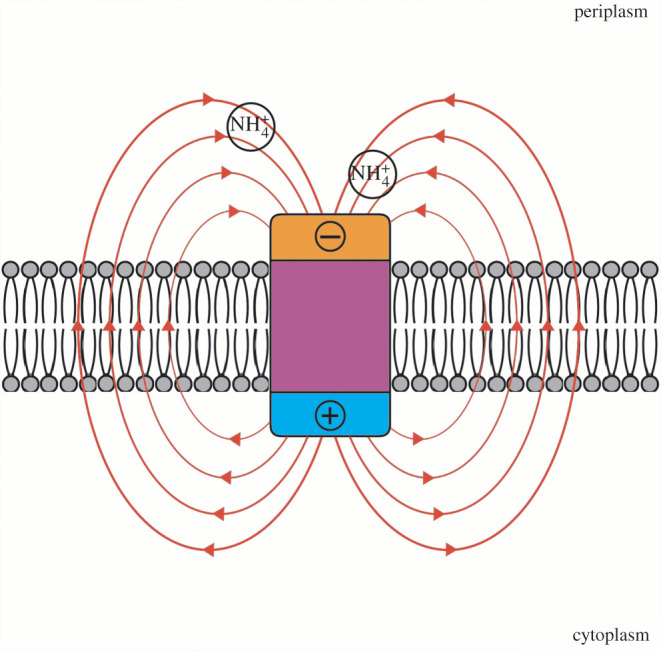
The electric field around Amt facilitates NH_4_^+^ uptake. The direction of the electric field flows outward from the positively charged end in the cytoplasm, loops around and exits the cell through the surrounding membrane space, then loops back inward towards the negatively charged end in the periplasm. Importantly, this force passes through the periplasm fluid and is thought to both recruit a higher proportion of cations to the periplasm vestibule while inhibiting anion binding, as well as help guide and orient NH_4_^+^ towards the binding pocket [16,25,29,36]. The hydrophobic properties of the transmembrane channel which requires deprotonation of NH_4_^+^ and subsequent conductance of NH_3_, selects against the passage of cations that would require replacement for their hydration shell, such as K^+^ [16].

Amt homologues can be classified by the presence or absence of a cleavable N-terminal signal peptide [32], which we indicate here with ‘+’ and ‘−’ symbols. ‘Amt+’ possess a cleavable N-terminal signal peptide for translocation into the cytoplasm membrane via the general secretory (Sec) pathway [19]. ‘Amt−’ do not possess the cleavable N-terminal signal peptide and have been suggested to translocate into the cytoplasm membrane via a non-classical secretion pathway [32]. Prokaryotes often encode and express multiple Amt paralogues, indicating subfunctionalization related to optimal function in different substrate concentrations and/or environmental pH [18,27,32–34]. Results from these previous experiments suggest that Amt− has a lower affinity and is used by microbes in higher [NH_4_^+^] environments. For both sets of Amt homologues, amino acid distribution is such that cytoplasm extensions have a higher net positive charge (e.g. rich in arginine, histidine and lysine amino acids) compared to periplasm extensions (rich in aspartic acid and glutamic acid), following a ‘positive-inside’ rule of transmembrane proteins [25,26,29,35]. This arrangement creates a dipole moment over the length of the protein. The oppositely charged transporter ends generate an electric field that, when interacting with a positively charged ion, exerts a force that loops externally around the protein (figure 1) and facilitates transport (approx. 2000 Debye for *E. coli* AmtB trimer [29]).

Microbial nutrient uptake alters concentrations of free substrates, making the process an important nutrient sink in biogeochemical cycles [37]. NH_4_^+^ is a key component of the nitrogen cycle and a prime example of a nutrient highly influenced by microbes who use it both as an anabolic N source and as an energy source (i.e. ammonia-oxidizing microorganisms). NH_4_^+^ exists in equilibrium with NH_3_ (H_2_O + NH_3_ ↔ OH^−^ + NH_4_^+^). At surface ocean pH, temperature, and salinity, approximately 80% of their combined total exists as NH_4_^+^ and increases as these parameters change with depth [38]. NH_4_^+^ concentrations in the open ocean peak at the base of the sunlit euphotic zone (>150 nM) then rapidly decrease to vanishingly low concentrations with depth (<5 nM), eventually reaching the limits of direct measurement [39–42]. Low NH_4_^+^ concentrations have previously been interpreted to indicate diminished relative importance as a nutrient and energy source in the deep ocean [43]. Instead, low concentrations reflect the balance between uptake and supply, with high-affinity NH_4_^+^ uptake balancing supply. Properties of Amt transporters that impart a competitive advantage at lower concentration environments may help explain the fate of NH_4_^+^ in the deep ocean.

Here, we use a global marine metagenomic dataset (Tara Oceans [44]) combined with a time-series dataset (Hawai'i Ocean Time-series at Station ‘A Long-term Oligotrophic Habitat Assessment’ (HOT/ALOHA) [10]) to compare the distribution of Amt sequence types across ocean depths, working under the assumption that deeper depths represent more NH_4_^+^ limited environments. We focus on three microbial taxa—Marine Group I (MGI) Archaea (formerly Thaumarchaeota and currently proposed as Class Nitrososphaeria within the Thermoproteota [45]), the gammaproteobacteria SAR86 (clades A, B and E), and the alphaproteobacteria SAR11—that are abundantly represented in both shallow and mesopelagic layers of the water column, as well as globally dispersed [44,46,47]. Little is known of the NH_4_^+^ affinity of these taxa, owing to the challenge of cultivating relevant representatives from the ocean and the difficulty with determining transport affinities at low substrate concentrations. Insight into their Amt protein sequence properties that conveys relative NH_4_^+^ affinity could reveal different competition strategies in different ocean layers.

## 2. Results

**Figure 2.**
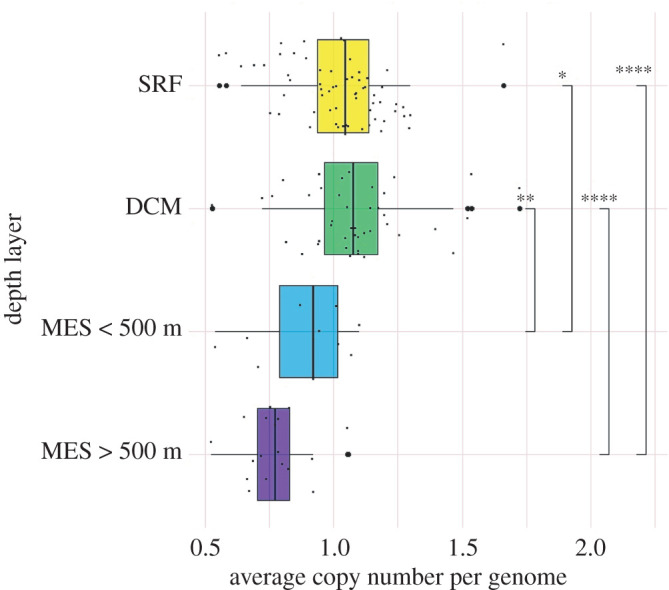
Average microbial *amt* gene copy number per genome decreases with depth in the ocean. Tara samples are separated into four separate depth layers: SRF (Tara SRF and MIX; yellow), DCM (Tara DCM; green), MES < 500 m (Tara MES samples from less than 500 m; blue) and MES > 500 m (Tara MES samples from greater than 500 m; purple). Individual Tara samples are plotted as black points surrounding their corresponding depth layer box plot. To aid in visualization of individual samples, points are offset from their gridlines and outlier samples are marked with a larger bold point on the gridline at their *x*-axis position. Wilcoxon–Mann–Whitney test comparisons and their *p*-values are displayed as brackets with corresponding significance ranges (*≤0.05, **≤0.01, ***≤0.001, ****≤0.0001).

Restricting Amt sequence comparisons to within each taxon and Amt type (Amt+ or Amt−) reduced the diversity of amino acid multiple alignment positional homology and facilitated direct comparison between ‘shallow’ (Tara samples labelled surface, deep chlorophyll maximum and mixed; HOT/ALOHA ≤175 m) and ‘deep’ (mesopelagic; HOT/ALOHA greater than 175 m) sampled sequences. Our depth boundary of 175 m in HOT/ALOHA samples separates the euphotic zone (shallow) from the non-euphotic zone (deep) [48]. The average copy number of Tara Oceans *amt* sequences relative to prokaryotic single-copy genes decreases with depth in our dataset, with medians ranging from 1.04 copies per genome in surface waters to 0.92 copies per genome below 175 m and decreasing further to 0.77 in samples from below 500 m (figure 2). However, these calculations assume that the diversity of microorganisms and their genomes were sampled to the same extent in all of the collected metagenomes, and therefore our copy number results warrant further validation.

**Figure 3.**
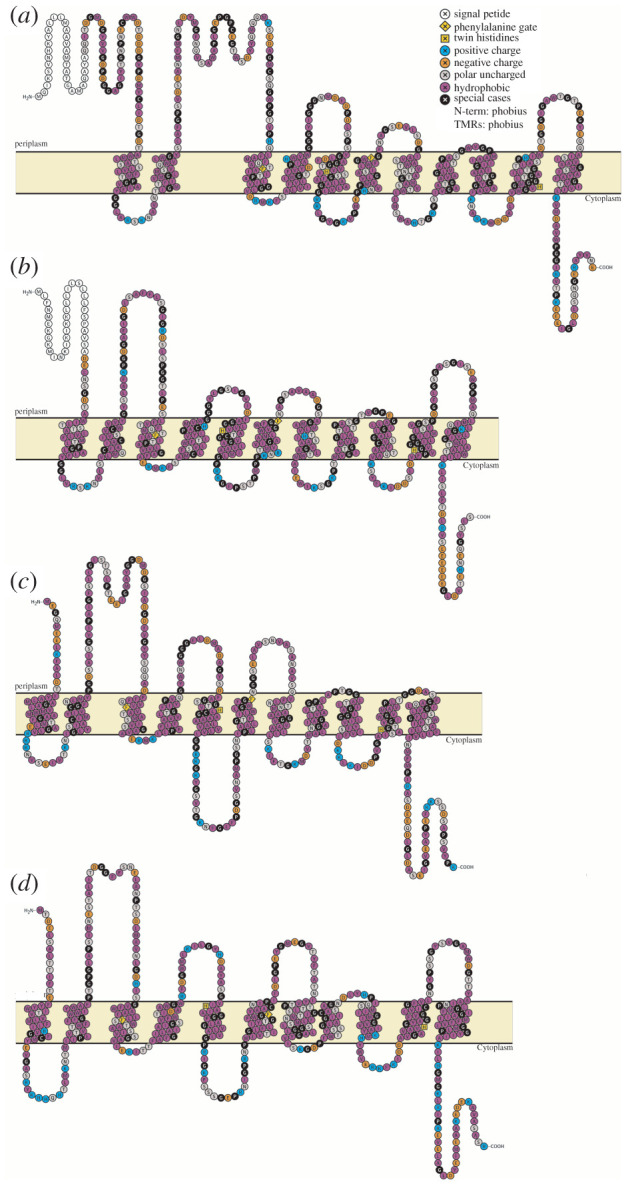
Transmembrane topologies of Amt from the three taxa examined in this study. (*a*) MGI Amt+ consensus sequence, (*b*) SAR86 Amt+ consensus sequence, (*c*) SAR86 Amt− consensus sequence and (*d*) SAR11 Amt− consensus sequence. Individual amino acids are labelled with their single letter code and colour coded based on side chain properties or their presence in well-characterized sequence motifs (Amt+ signal peptide, white; phenylalanine gate conserved motif, yellow diamond; twin histidine conserved motif, yellow square; positively charged, blue; negatively charged, orange; polar uncharged, grey; hydrophobic, purple; special cases, black). The cytoplasmic membrane is represented as a yellow band that separates the periplasm (top) and cytoplasm (bottom), with periplasm extensions, cytoplasm extensions and transmembrane regions mapped in their respective regions.

**Figure 4.**
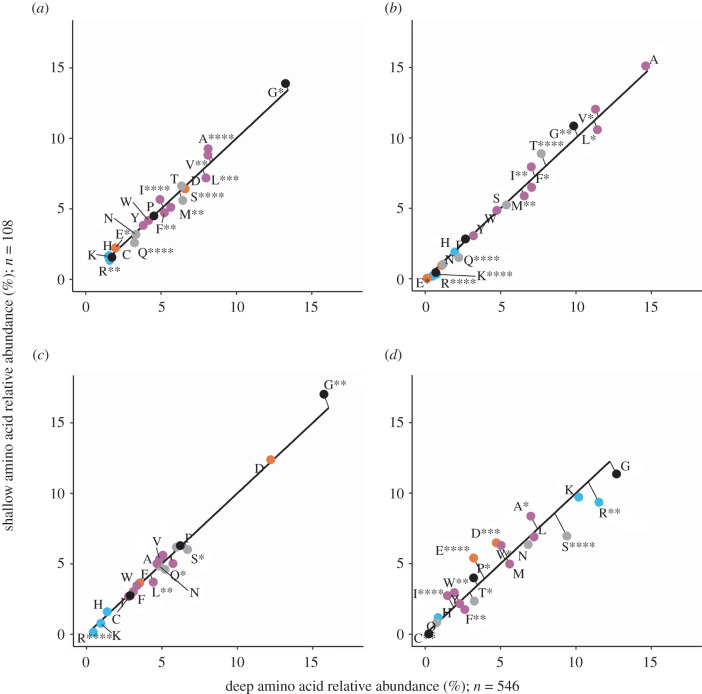
Amino acid usage for different regions of MGI Amt+ sequences: (*a*) overall protein, (*b*) the membrane region, (*c*) the periplasm region and (*d*) the cytoplasm region. Each graph shares the same axes of amino acid relative abundance, with deep sequences on the *x*-axis and shallow sequences on the *y*-axis. The black line represents the expected amino acid abundances if there were no difference in usage between shallow and deep sequences. The points are actual amino acid usage labelled with conventional amino acid single letter codes and coloured by side chain properties, positive (blue), negative (orange), uncharged (grey), hydrophobic (purple) and black (special cases). Points that fall below the expected line are more abundant in deep sequences than in shallow sequences. Plotted points that fall above the expected line are more abundant in shallow sequences than in deep sequences. Significance in the deviation from the expected line was calculated with chi-square analysis, *≤0.05, **≤0.01, ***≤0.001, ****≤0.0001.

**Figure 5.**
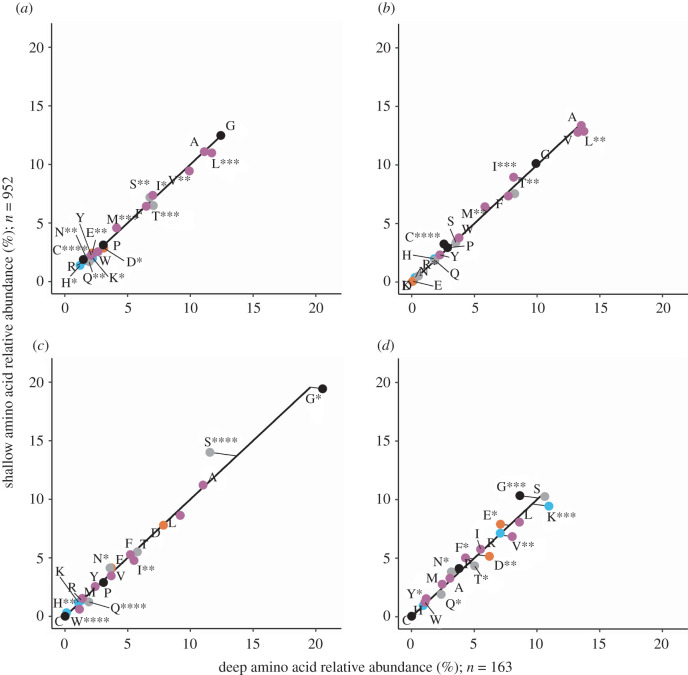
Amino acid usage for different regions of SAR86 Amt+ sequences. Panels, axes and points are as in figure 4.

**Figure 6.**
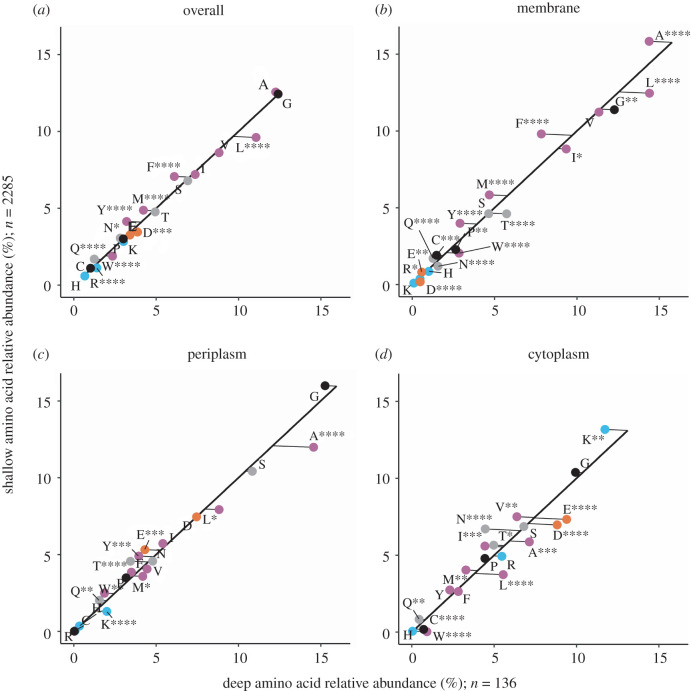
Amino acid usage for different regions of SAR86 Amt− sequences. Panels, axes and points are as in figure 4.

**Figure 7.**
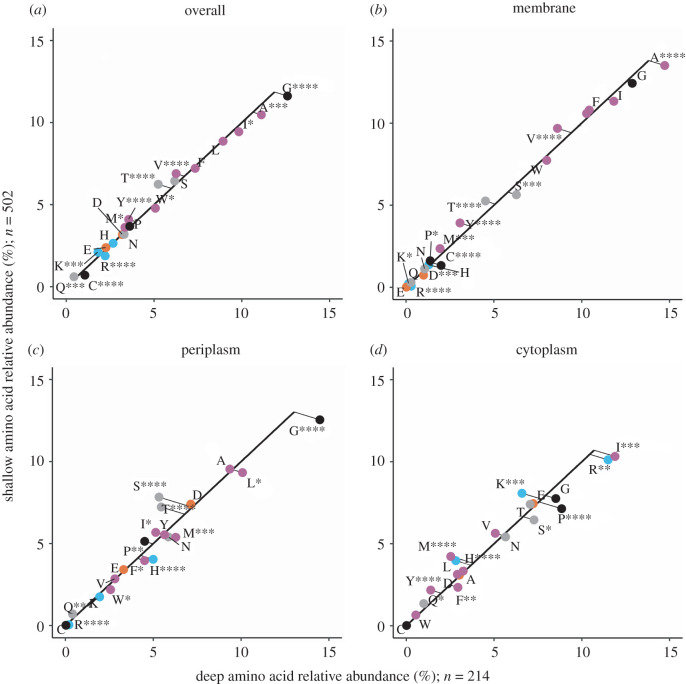
Amino acid usage for different regions of SAR11 Amt− sequences. Panels, axes and points are as in figure 4.

We compared four distinct Amt variants from shallow and deep ocean sample metagenomes: a thaumarchaeal Amt+, both an Amt+ and Amt− from SAR86, and Amt− from SAR11. MGI Amt− and SAR11 Amt+ clades were not abundant enough in both sample depth layers to include for analysis. For each of the Amt comparisons, we created multiple sequence alignments of Amt and used the resulting consensus sequences to create secondary structure topology maps (figure 3*a–d*), which defined Amt sequence localizations and allowed us to perform Chi-square analysis on amino acid compositions (figures 4–7). For the overall Amt alignments, the relative abundances of charged amino acids were low when compared to polar uncharged and hydrophobic amino acids (figures 4*a*, 5*a*, 6*a* and 7*a*). Overall Amt amino acid usages were greatly influenced by the membrane-bound regions, which are composed mostly of polar uncharged and hydrophobic amino acids (figures 4*b*, 5*b*, 6*b* and 7*b*). In all of our Amt comparisons, the relative abundances of charged amino acids in the periplasm and cytoplasm extensions exhibit the ‘positive-inside’ rule discussed above, where the net charge of cytoplasm extensions is more positive than the net charge of periplasm extensions. However, for each of our comparisons, the degree to which the rule is followed differs between shallow and deep samples.

The charged amino acid composition of periplasm extensions for both of our Amt+ alignments are similar between shallow and deep samples (table 1; figures 4*c* and 5*c*), but cytoplasm extensions from deep samples have an increased positive charge relative to shallow samples (table 1; figures 4*d* and 5*d*). When calculating normalized net charges for cytoplasm extensions at pH 7, a value generally relevant to microbial cytoplasm, MGI Amt+ sequences from deep samples have a charge that is approximately 6.6 charge units more positive than in shallow samples (table 1; calculation described in Methods). In SAR86 Amt+ sequences, cytoplasm extensions have a charge that is approximately 1.2 charge units more positive in deep samples than in shallow samples (table 1). If we then subtract periplasm net charges from cytoplasm net charges to calculate a dipole charge difference, MGI Amt+ sequences from deep samples have a dipole charge difference that is approximately 5.8 charge units greater than in shallow sample sequences (table 1). SAR86 deep sample Amt+ sequences have a similar but less pronounced charge difference that is approximately 1.0 charge units greater than in shallow samples (table 1).

**Table 1.** Normalized net amino acid charge distributions across periplasm and cytoplasm consensus sequence regions of MGI Amt+, SAR86 Amt+, SAR86 Amt− and SAR11 Amt− from deep and shallow samples. Net charges are calculated from charged amino acid relative abundances as described in the methods and dipole charge difference is defined as: cytoplasm net charge – periplasm net charge. Chi-square tests of homogeneity on the periplasm and cytoplasm charged amino acid distributions in all four deep and shallow sample comparisons have *p-*values < 0.001.

	periplasm net charge	cytoplasm net charge	dipole charge difference
MGI Amt+			
deep	−14.22	13.88	28.1
shallow	−14.96	7.31	22.27
difference	0.74	6.57	5.83
SAR86 Amt+			
deep	−9.16	4.81	13.97
shallow	−9.36	3.6	12.96
difference	0.2	1.21	1.01
SAR86 Amt−			
deep	−9.65	−1.03	8.62
shallow	−11.4	3.81	15.21
difference	1.75	−4.84	−6.59
SAR11 Amt−			
deep	−7.8	8.11	15.91
shallow	−8.61	8.06	16.67
difference	0.81	0.05	−0.76

In comparison to the Amt+ type, patterns of charged amino acid variation with depth were different for the Amt− we examined (table 1; figures 6 and 7). In SAR86 Amt− sequences (figure 6), most of the net charge variation again occurs in cytoplasm extensions but also with some difference in periplasm extensions. Cytoplasm extensions in SAR86 Amt− from deep samples are approximately 4.8 charge units less positive than shallow samples, and periplasm extensions are approximately 1.8 charge units more positive in deep samples (table 1). If we subtract periplasm net charges from cytoplasm net charges, SAR86 Amt− sequences from deep samples have a dipole charge difference that is approximately 6.6 charge units less than shallow sample sequences (table 1). In SAR11 Amt− sequences, most of the net charge difference between shallow and deep samples occurs in periplasm extensions, which are approximately 0.8 charge units more positive in deep samples than in shallow samples (table 1). SAR11 Amt− sequences have a similar but less pronounced dipole charge difference between oppositely facing extensions to that of SAR86 Amt−, with a value that is approximately 0.8 charge units greater in shallow samples than in deep samples due to minimal cytoplasm extension differences (table 1).

In addition to the charge differences described above, each Amt comparison differs in the counts of sequences from shallow or deep samples (figures 4–7), the magnitude of the dipole charge difference between shallow and deep samples (e.g. 22.27 and 28.1 charge units for MGI Amt+, respectively; table 1), and the utilization of amino acids that amount to net charge differences (figures 4–7). Each Amt in our comparisons utilizes a different distribution of charged amino acids while maintaining cytoplasm extensions that are more positive than periplasm extensions. Chi-square tests of homogeneity on the periplasm and cytoplasm charged amino acid distributions in all four deep and shallow sample comparisons have *p*-values < 0.001, indicating amino acid abundances of deep and shallow Amt sequences are composed of different amino acid profiles.

## 3. Discussion

**Figure 8.**
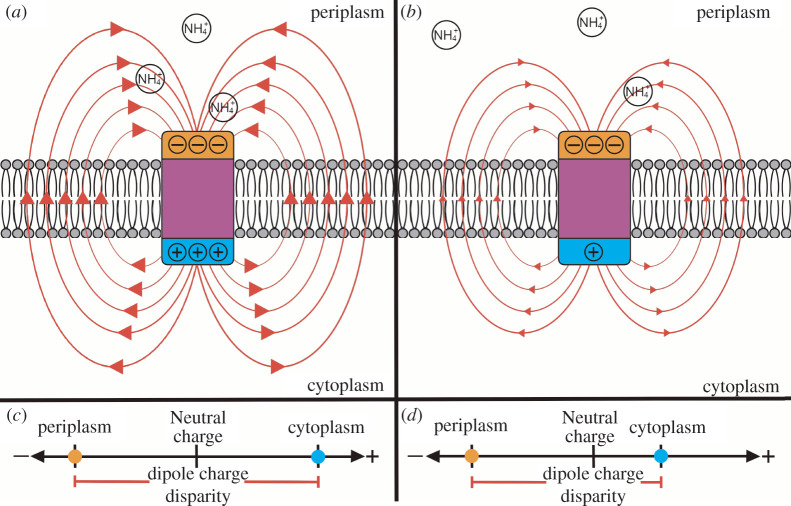
Schematic of Amt electric fields created by dipole amino acid charge distributions. The charge disparity of Amt is shown between the positively charged cytoplasm (blue) and negatively charged periplasm (orange) regions, separated by the hydrophobic membrane (purple) region and lipid bilayer. With equal periplasm charges, a large dipole charge disparity created by a strongly positive cytoplasm charge (*a*) and small dipole charge disparity created by a less positive cytoplasm charge (*b*) are compared to visualize the effects on the electric field. Representations of large and small charge disparities between periplasm and cytoplasm regions are plotted along an axis in (*c*) and (*d*), respectively, with the membrane region not plotted. Red arrow lines show the direction and approximate paths of the electric field as they would act on positively charged ions. Positively charged ammonium ions in the periplasm are depicted to be attracted towards the Amt periplasm region by the electric field. For simplicity, any electric field paths that flow through the Amt membrane region are not depicted, since the strongly hydrophobic properties of the membrane region would inhibit the travel of charged ions, and thus the influence of the field.

Our study aimed to determine whether or not functional adaptations to low NH_4_^+^ concentrations could be discerned from sequence data, using the Amt transporter as a test case. We did not identify any Amt amino acid sequence regions that are specific to shallow or deep samples in any of the lineages within our focus. However, we did observe differences in the Amt amino acid usage between sequences from shallow and deep samples. By increasing the proportion of negatively charged amino acids in the periplasm, organisms could theoretically increase the Debye strength of the electric field surrounding Amt, increasing the effective range of their attractive force and thereby their substrate affinity [29,49]. Likewise, our observation of increasing the positive charges of MGI and SAR86 Amt+ cytoplasm extensions would have a similar effect, increasing the charge disparity of the dipole and thus the effect of the electric field. The strength of an Amt electric field would depend on the magnitude of the dipole charge disparity (figure 8), with stronger Amt electric fields possibly increasing NH_4_^+^ acquisition from the periplasm. Additionally, the trimer quaternary structure of Amts further strengthens the attractive force in the periplasm with an additive effect [25], meaning increases in monomer electric field strength would be amplified.

It may seem counterintuitive for a transmembrane transporter protein to have higher substrate affinity by adaptation of regions facing away from substrate but it is an elegant solution to increasing NH_4_^+^ recruitment without altering substrate binding and deprotonation environments. Like many proteins, the binding pocket size, shape and charge of Amts at the periplasm mouth of the transmembrane region is a critical component of NH_4_^+^ recruitment and specificity [8,36]. Maintaining the amino acid tertiary structure of the Amt binding site may be an evolutionary barrier that impedes large changes in periplasm extension amino acid usage.

Within the two Amt+ containing taxa that we focused on, the ammonia-oxidizing MGI had the greatest charge disparity from shallow to deep ocean samples and the difference in SAR86 Amt+ was significant but relatively minimal (table 1). This could be due to greater selective pressure for attracting NH_4_^+^ for catabolic demands compared with solely biosynthetic needs. Higher affinity Amts would also help marine microbes, especially MGI, meet NH_4_^+^ influx demands to offset the loss of NH_3_ due to high membrane permeability and a concentration gradient that favours diffusion out of the cell [14]. If permeation of NH_3_ is great enough in MGI, it may create localized NH_3_ concentrations for oxidation by membrane-bound ammonium monooxygenase [50]. If Amt+ sequences in the deep ocean are exhibiting our observed exaggerations of dipole charge in response to competitive evolutionary forces, then that would indicate NH_4_^+^ in the deep ocean is in high demand and its scarcity is due to efficient uptake by microbes expressing such Amts. Similarly, if a large Amt dipole charge exaggeration is the result of competition for a limited amount of NH_4_^+^, then a small dipole charge exaggeration (as seen in SAR86 Amt+) could mean that environmental NH_4_^+^ concentration is not a strong selection factor for growth or that these organisms rely on the transport of other anabolic sources, such as dissolved organic matter [51,52]. A stronger electric field may even become inhibitory if it over-attracts substrate (toxicity and decreased flux [13,53]) and non-substrate (competitive inhibition [27]). Alternatively, a large dipole charge exaggeration may allow MGI to compensate for the scarcity of energy in the deep ocean, which limits the amount of Amts (and total proteins) that can be produced by MGI cells to increase NH_4_^+^ uptake [54].

In culture, *Nitrosopumilus maritimus* SCM1 (MGI) Amt− transcripts were reduced during NH_4_^+^ starvation (nM concentration), while Amt+ transcripts were unchanged [34]. *N. maritimus* SCM1 are known to be well-adapted to acquiring NH_4_^+^ in low substrate environments, having a high affinity with a Michaelis constant (*K*_m_) of 132 nM total NH_4_^+^ [33]. Our low counts of MGI Amt− sequences (too low for Chi-square analysis) from deep samples are consistent with findings that Amt− may not be present at all in deep ocean MGI [55], despite being present in genomes from cultured MGI [56,57].

The charge distributions that we observe for the two Amt− containing taxa have an opposite direction of exaggeration than the Amt+ containing taxa. That is, they both have an increased dipole charge distribution in shallow sample sequences relative to deep samples. A low affinity transporter (Amt−) might not be expected to evolve higher affinity variants in low substrate conditions, such as the deep ocean if a high-affinity transporter (Amt+) is already encoded within the genome. The Amt− dipole charge difference that we observed is most prominent in SAR86, with members of the SAR11 clade having the smallest difference between deep and shallow samples (minimal difference but significant; table 1). Under our competition premise, an increased dipole charge distribution in shallow sample sequences relative to deep would mean that NH_4_^+^ acquisition is more of a limiting factor for growth in the shallow ocean for these taxa than in the deep. Since NH_4_^+^ concentration decreases with depth, this suggests that SAR11 and SAR86 in the deep ocean are limited by some factor other than NH_4_^+^. At least some clades of SAR11 and SAR86 are capable of proteorhodopsin-conferred photo-heterotrophy, using light to generate ATP and enhance nutrient uptake for growth [51,58–61]. Without this energy-harvesting process available in the deep ocean, the chemoheterotrophic SAR11 and SAR86 could be limited by organic carbon availability, not nitrogen. It may even be disadvantageous to have higher affinity Amts (both Amt− and Amt+) that spend limited energy bringing in excess NH_4_^+^ that cannot be used for growth. Moreover, increased affinity does not necessarily equate to increased NH_4_^+^ uptake rate. Optimal uptake kinetics models imply a trade-off between increased affinity and maximum uptake velocity for whole cells, which may mean that these organisms increase membrane transporter density or deplete intracellular NH_4_^+^ to free enzyme active sites as means for increased uptake [62].

SAR86 also appears to be more dependent on NH_4_^+^ acquisition than SAR11. So far, no urease genes have been found in SAR86 genomes [63], but have been found in SAR11 [64]. SAR86 genomes also have low counts of ABC-type transporter genes that could be utilized for amino acid uptake [51], uptake that was indeed measured to be low in ocean waters [65]. By contrast, SAR11 genomes contain many amino acid transporter genes that have high affinity and are multifunctional [66,67], and SAR11 are responsible for up to 50% of amino acid assimilation in ocean surface waters [68]. SAR11's ability to efficiently scavenge N from multiple sources could be why there is little difference between shallow and deep sample sequences. For SAR86, the combination of a high-affinity Amt+ and a low-affinity Amt− with an exaggerated dipole in shallow samples could both be needed to meet N requirements.

The high abundance of lysine and aspartic acid that we see in SAR86 Amts (figures 5 and 6) could be a result of necessity, since SAR86A may be deficient in arginine and histidine with an excess of aspartic acid. SAR86A, a clade of SAR86, lack proteins required for histidine and arginine synthesis pathways which utilizes aspartic acid as a precursor [51]. In marine microorganisms with streamlined genomes, there is a trend of increased compositional bias for nucleotide and amino acids of lower N content, such as the substitution of lysine for arginine [9,69]. Lower N content compositional bias is thought to reduce the amount of N needed for cell growth and replication [70], but further study is needed to show the extent of this trend specifically in SAR86 clades. In our study, SAR86A represented approximately 8% of SAR86 Amt− shallow sequences, approximately 4% of SAR86 Amt− deep sequences, approximately 10% of Amt+ shallow sequences, and 0% of Amt+ deep sequences. These percentages are not definite, since approximately 73% of Amt+ and approximately 91% of Amt− SAR86 annotations were not clade specific. However, it is interesting that Amts in SAR86A maintain the ‘positive-inside’ rule and appear to evolve a charge exaggeration even while auxotrophic for some charged amino acids, implying that charge properties of Amt must be crucial for function.

While we are unable to quantify actual changes in the dipole moment strength among Amt variants, which would require crystal structure tertiary coordinates of charged amino acids in order to be calculated [49], we are able to make qualitative comparisons of dipole distribution differences between taxa, ocean layers and Amt types. In all clades, the major differences in amino acid distribution occur in the cytoplasm extensions, which are collectively more positively charged than their periplasm extensions. An interesting exception is the cytoplasm-facing C-terminus tail, which has a net negative charge [19]. This negatively charged tail is modelled to interact with positively charged cytoplasm extensions of neighbouring Amt monomers, linking them into their trimer quaternary structure as well as facilitating the binding of regulatory GlnK or GlnK-like proteins [18,19,71,72]. When C-terminal tails are omitted from our analysis to confirm that their absence in partial sequences are not the sole cause of our observations, the observed disparity between deep and shallow net charges are altered but not negated. Intriguingly, only Amt− have an extended C-terminal tail and an adjacent *glnK* gene in the genomes of ammonia-oxidizing archaea [32]. If similar features hold true for Amt− of other taxa, it could mean that Amt− are more likely to have a lesser dipole disparity than Amt+ due to the negative charge of extended C-terminal tails.

To speculate, over evolutionary time, Amt electric fields could be tunable to specific NH_4_^+^ concentrations and/or ecological conditions; possessing a transporter that is properly tuned to environmental conditions offers a competitive advantage. Possessing multiple variants of Amt in a genome may be advantageous for populations that need to regulate NH_4_^+^ transport in fluctuating conditions [32]. Our results suggest a stronger Amt electric field helps competing populations only if NH_4_^+^ acquisition is limiting growth. Other membrane transporter proteins could utilize similar electric field strategies to compete for growth-limiting ion substrates. Our work will inform future examination of dipole charge distribution with electric field strength and substrate affinity measurements, testing the expectations of exaggerated dipole charge distribution leading to increased NH_4_^+^ recruitment.

## 4. Methods

We used data from 243 Tara Oceans [44] and 165 HOT/ALOHA metagenome samples [10]. We found 32 566 partial and complete predicted protein-coding *amt* sequences from pre-existing metagenome unbinned contig assemblies (electronic supplementary material, file S1) with a DIAMOND (v. 0.9.24.125) [73] blastx search against a curated set of 52 bacterial and archaeal UniProt [74] Amt sequences, with an E-value cutoff of 0.001. Taxonomy classifications of the *amt* sequences were assigned with a DIAMOND blastx search, E-value cutoff of 1 × 10^−20^, against the NCBI non-redundant protein database. The annotated predicted protein-coding *amt* sequences were translated to Amt amino acid sequences with TranslatorX [75]. The Amt sequences were then clustered with CD-HIT (v. 4.8.1) [76] iteratively for clustering cutoffs of 0.5 to 1.0 to confirm sequence grouping by taxonomy annotation, which can have varying levels of conservation between taxa. Within our whole Amt dataset, approximately 75% of Amt sequences are from Tara samples. Within the three taxa of our focus, approximately 80% of Amt sequences are from Tara samples.

*amt* average copy numbers per genome in the water column were estimated by comparing *amt* abundances in our Tara Oceans metagenome search data relative to 10 prokaryotic single-copy genes suited for metagenomic applications (COG0012, COG0016, COG0018, COG0172, COG0215, COG0495, COG0525, COG0533, COG0541, COG0552) [77], using a DIAMOND E-value of 1 × 10^−20^ and UniProt single-copy gene sequences. The number of genomes in each metagenome was estimated by calculating the average number of protein sequences assigned to the 10 prokaryotic single-copy COGs. The average number of *amt* sequences per genome was calculated by dividing the number of collected *amt* sequences by the estimated number of genomes. *amt* average copy numbers per genome were plotted in R (ggplot2) [78]. For these calculations, water column samples were separated into four depth layers, surface and mixed layer samples (Tara SRF and MIX), deep chlorophyll maximum (DCM) samples, mesopelagic (MES) samples < 500 m, and MES greater than 500 m. Wilcoxon–Mann–Whitney tests with post hoc Bonferroni adjustments for multiple comparisons were used to test *amt* average copy number depth layer comparisons for significance in R [79].

We then investigated Amt sequences separated into ‘shallow’ (Tara SRF, MIX, DCM, and HOT/ALOHA ≤175 m) and ‘deep’ (Tara MES and HOT/ALOHA > 175 m) ocean layers looking for conserved amino acid positions or patterns in their sequence alignments (MAFFT v. 7.407) [80] that correspond to sample depth, as well as amino acid composition. Through multiple alignment of MGI, SAR11 and SAR86 variant clades and secondary structure prediction (Protter v. 1.0) [81] of consensus sequences, we isolated our analysis to specific regions of the protein sequences: periplasm-facing extensions (excluding the N-terminal signal peptide region in Amt+), cytoplasm-facing extensions, and membrane-bound regions. Protter secondary structure prediction of consensus sequences utilizes Phobius [82] to predict signal peptides and transmembrane regions. To avoid extending consensus sequences with residues that do not reflect the properties of the whole Amt variant clade, multiple alignment columns were discarded from the consensus sequences if they had low representation, with less than 5% of the aligned sequences containing a residue at the removed column position. Removed columns are marked as ‘NA’ in the ‘Threshold column counts' tabs of electronic supplementary material, files S2–S5. We then calculated the relative abundance of amino acids per sequence. A chi-square test of homogeneity between shallow and deep sample abundances of amino acids, where the null hypothesis assumes identical distribution, was used to determine if the distributions of cytoplasm-and periplasm-extension charged amino acids (arginine, histidine, lysine, aspartic acid and glutamic acid) differed, confirming two separate groups of sequences with all comparisons indicating a *p*-value < 0.001 (electronic supplementary material, files S2–S5). Relative abundance of individual amino acids per total amino acids for shallow and deep sequences within each region were compared and plotted in R (ggplot2). Chi-square goodness of fit was used to test the significance of individual amino acid relative abundances per total amino acids, where the null hypothesis assumes no difference in relative abundance between shallow and deep Amt sequences as the expected outcome (electronic supplementary material, files S2–S5).

Net charges were calculated from the percentages of each charged amino acid in cytoplasm and periplasm regions to normalize count information in both shallow and deep samples. At pH 7, arginine, histidine, lysine, aspartic acid and glutamic acid have charges of approximately 1, 0.1, 1, −1 and −1, respectively [83]. These amino acid charges were multiplied by their normalized percentages in periplasm and cytoplasm regions for both shallow and deep samples, yielding normalized net charges and differences that are summarized in table 1.

## Data Availability

All original sequence data are public data and have been previously published elsewhere. Sequences that were used for this work as a result of alignment searching and all spreadsheets that contain amino acid counts and calculations are available as electronic supplementary material [84].
