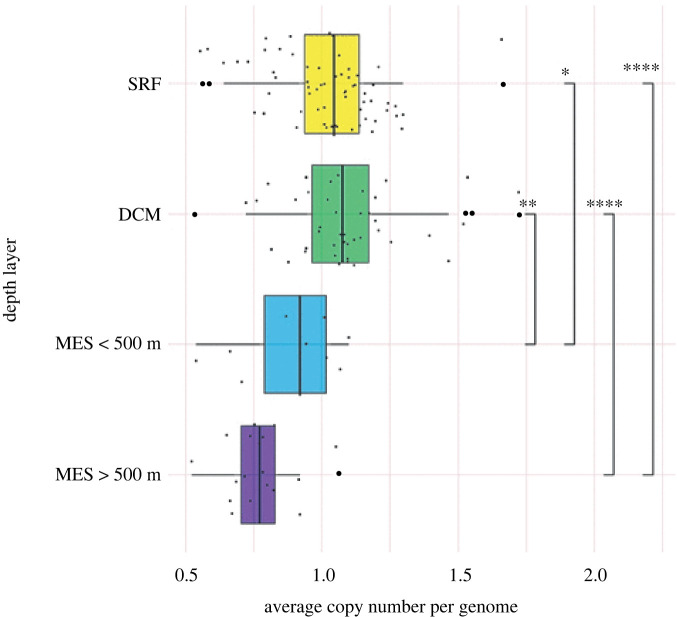# Correction to ‘Exaggerated trans-membrane charge of ammonium transporters in nutrient-poor marine environments’ (2022) by Kellom *et al*.

**DOI:** 10.1098/rsob.220268

**Published:** 2022-09-28

**Authors:** M. Kellom, S. Pagliara, T. A. Richards, A. Santoro

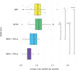

*Open Biology* **12**, 220041. (Published online 13 July 2022) (https://doi.org/10.1098/rsob.220041)

In figure 2, a typo of ‘MES > 500 m’ was introduced to the third *y*-axis label from the top when edited to meet house style. This label has been corrected to ‘MES < 500 m’ in the updated version of figure 2, below.

**Figure:**